# Correspondence: Response of a gravimeter to an instantaneous step in gravity

**DOI:** 10.1038/s41467-017-01348-z

**Published:** 2017-10-17

**Authors:** Thomas H. Heaton

**Affiliations:** Division of Geological and Planetary Sciences, Caltech, Pasadena, CA 91125 USA

## Introduction

Montagner et al.^1^ presented evidence that a signal observed prior to P-waves on gravimeters operating in Japan during the 2011 Tohoku-Oki earthquake was caused by gravitational field changes propagating at light speed. In this comment, I explore the expected response of gravimeters attached to the Earth’s surface and subjected to an instantaneous acceleration step, $g\left( t \right) = {g_0} + \Delta gH\left( t \right)$, where $H\left( t \right)$ is a Heaviside step function. I argue that at the time of the step, inertial accelerations from the elastic response of the Earth exactly cancel the gravitational step; in order to observe the gravity step using a gravimeter, one must wait until the Earth deforms and begins to re-equilibrate. This conclusion is an application of the principle of equivalence^2^ between gravitational and inertial mass. In the case of the signal reported^1^ for Tohoku-Oki, I estimate that inclusion of the Earth’s elastic response should significantly decrease the expected acceleration at the time of the P-wave.

To determine a complete solution for the response of a gravimeter attached to the Earth’s surface, I would need to (1) calculate the spatial distribution of gravitational changes caused by mass redistribution in an earthquake, (2) calculate the motion at the gravimeter due to unbalanced elastic and gravitational forces, and (3) sum the gravity change with the inertial accelerations determined in step 2. Finding the Earth’s elastic response is a complex problem that can be approached by summing the responses of the Earth’s normal modes. When the Earth response problem is decomposed into a sum of modes, the time response of each mode simplifies into a set of equivalent linear single-degree-of-freedom (sdof) oscillator problems driven by a step function forcing function of the appropriate amplitude. Alternatively, I could use a finite-element code to calculate both the change in gravity and also the motion of the Earth caused by those changes in gravitational body forces. Performing those calculations would transform this brief communication into a long treatise. However, I can provide insight into this problem by introducing a whimsical gedanken experiment.

Consider the response of a gravimeter attached to the Earth’s surface, and that the gravitational constant is suddenly transformed to zero, or $\hat G = \left[ {1 - H\left( t \right)} \right]G$. You might guess that when this happened, we would all instantly become weightless. However, what would actually happen is that the Earth (previously compressed by gravity) would instantaneously begin to expand with an outward acceleration equal to gravity. That is, initially our weight would be virtually unchanged until the Earth’s outward acceleration decreased as it started to approach its new uncompressed weightless state; only then would we truly feel weightless.

This gedanken experiment illuminates the basic principle of my comment about using gravimeters to detect changes in gravity. Gravimeters are vertical accelerometers that measure the sum of the vertical acceleration of the Earth’s surface plus the vertical gravitational acceleration. When the Earth is in gravitational/elastic equilibrium, then there is no inertial acceleration of the Earth and then the gravimeter measures only the gravitational acceleration.

**Fig. 1:**
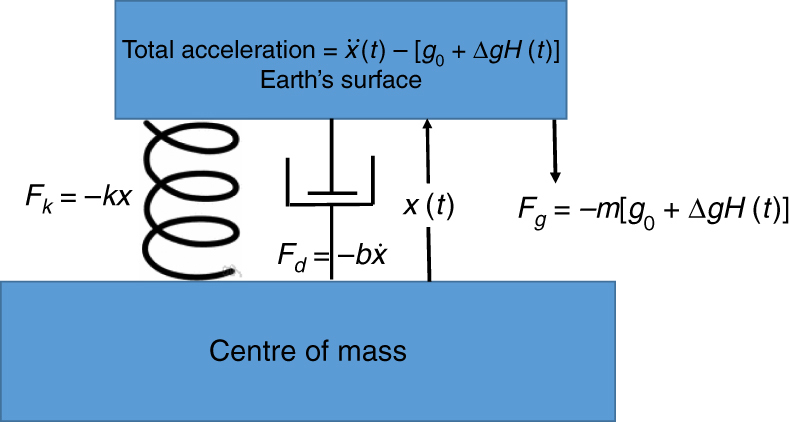
Schematic showing the parameters used to describe the acceleration of an elastically supported mass in the presence of a downward gravitational acceleration with an instantaneous step of $\Delta g$. $x$ is upward motion with respect to elastic/gravitational equilibrium

A more quantitative description of the dynamics of an elastic body that responds to gravitational changes comes from considering the case of a linear sdof oscillator that is attached to some stationary point; for a deformable body, it is the center of mass. The solution of this sdof provides the time history of motions of the Earth described by ${\,}_0{S_0}$ (Earth’s fundamental radial mode). It also provides a solution of the gedanken problem (at least for the first seconds when the response is linear). Figure 1 shows parameters used for this calculation. I assume that the downward gravitational force on $m$ is $mg\left( t \right) = m{g_0} + m\Delta gH\left( t \right)$, and that prior to the gravity change, the Earth is in static equilibrium, and that the Earth’s elastic response is linear for small perturbations from equilibrium.

The equation of the generalized motion of the Earth’s surface is

1 $$\ddot x + 2\beta \dot x + \omega _0^2x = \Delta gH\left( t \right)$$

where $\omega _0^2 = k{\rm{/}}m$, and $\beta = b{\rm{/}}2m$. ${\omega _0}$ is the characteristic frequency at which the Earth oscillates about its new equilibrium (about 8.1 × 10^−4^ Hz), and $2\beta = \frac{{{\omega _0}}}{{\sqrt {\frac{1}{2} + {Q^2}} }} \approx \frac{{{\omega _0}}}{Q}$, where ${Q_{_{0}{S_0}}} \approx 5500$ for ${\,}_0{S_0}$. The motions for small times can be determined by recognizing that the third term in (1) is negligible at short times ($x$ is initially zero). That is, for short times,

2 $$\ddot x - \Delta gH\left( t \right) \approx - 2\beta \dot x$$

Since the system is initially at rest (i.e., equilibrium), $\dot x\left( {t = 0} \right) = 0$, and then $\ddot x - \Delta gH\left( t \right) \to 0 \, {\rm{ when }} \, t \to 0$. The full solution of (1) with at-rest initial conditions is

3 $$\begin{array}{l} 	 x\left( t \right) = \frac{{ - \Delta gH\left( t \right)}}{{\omega _0^2}}\left[ {1 - {e^{ - \beta t}}\cos \left( {{\omega _1}t} \right) - \frac{{\beta {e^{ - \beta t}}}}{{{\omega _1}}}\sin \left( {{\omega _1}t} \right)} \right] \\ 	 \approx \frac{{ - \Delta gH\left( t \right)}}{{\omega _0^2}}\left[ {1 - {e^{ - \beta t}}\cos \left( {{\omega _1}t} \right) - \frac{{{e^{ - \beta t}}}}{Q}\sin \left( {{\omega _1}t} \right)} \right]\\ \end{array}$$

where ${\omega _1} \equiv \sqrt {\omega _0^2 - {\beta ^2}}$. Taking time derivatives of (3),

4 $$\dot x\left( t \right) = \frac{{ - \Delta gH\left( t \right)}}{{{\omega _0}}}{e^{ - \beta t}}\sin \left( {{\omega _1}t} \right)$$

And

5 $$\begin{array}{l} 	 \ddot x\left( t \right) = \frac{{ - \Delta gH\left( t \right)}}{{{\omega _0}}}{e^{ - \beta t}}\left[ { - \beta \sin \left( {{\omega _1}t} \right) + {\omega _1}\cos \left( {{\omega _1}t} \right)} \right] \\ 	 \approx \frac{{ - \Delta gH\left( t \right)}}{{{\omega _0}}}{e^{ - \beta t}}\left[ {{\omega _1}\cos \left( {{\omega _1}t} \right) - \frac{1}{Q}\sin \left( {{\omega _1}t} \right)} \right]\\ \end{array}$$

If the damping is small $\left( {{\omega _0} \gg \beta } \right)$, then ${\omega _1} \approx {\omega _0}$ and

6 $$x\left( t \right) \approx \frac{{ - \Delta gH\left( t \right)}}{{\omega _0^2}}\left[ {1 - \cos \left( {{\omega _0}t} \right)} \right]$$

and

7 $$\ddot x\left( t \right) \approx - \Delta gH\left( t \right)\cos \left( {{\omega _0}t} \right)$$

The total vertical acceleration of the mass (inertial acceleration plus gravity) is then

8 $$\ddot x\left( t \right) - \Delta gH\left( t \right) \approx - \Delta gH\left( t \right)\left[ {1 - \cos \left( {{\omega _0}t} \right)} \right]$$

Notice that as $t \to {0^ + }$, the inertial response of the Earth, $\ddot x\left( t \right)$, exactly cancels the gravitational change $\Delta g$. This is because inertial accelerations from unbalanced elastic forces are indistinguishable from unbalanced gravitational accelerations. Notice that this cancelling effect is lessened if the damping is increased; there is a second term in (5) that increases as $\frac{1}{Q}\sin \left( {{\omega _1}t} \right)$. This term is from viscous forces that are not part of the static equilibrium problem, and this term grows linearly with time. If the Earth was heavily damped, then the gravitational forces would be easier to observe with an accelerometer. However, since $Q$ of the Earth is large (>500), this damping term is very small.

Assuming that the elastic response of the Earth is approximately given by ${\,}_0{S_0}$, I used (6) to estimate that the acceleration signal after 12 s would be about $2 \times {10^{ - 3}}\Delta g$. Even after 120 s, it only grows to $0.19\,\Delta g$.

Although this simple sdof gedanken problem is a gross oversimplification of the Earth’s response, it does allow us to guess that the acceleration response at a point will include the inertial effects of changing the gravimeter’s initial equilibrium position to a new equilibrium position appropriate for the gravity perturbation. The gravimeter will then oscillate about that new position until the motions damp out (from inelastic and radiation damping). Nevertheless, the initial acceleration of the gravimeter will be equal and opposite to the gravity change. Considering the Tohoku earthquake, there are only two plausible length/time scales to determine this oscillation period; the first is the Earth’s dimension (as in ${\,}_0{S_0}$), and the second is the source–observer distance. For the Tohoku earthquake, the travel time of elastic deformations between the source and observer seems to be the natural scale. I speculate that the effective period will be on the order of 500 s, which is four times the S-wave travel time. ${Q_S}$ for this deformation may be about 500. That is, I hypothesize that the net acceleration on a gravimeter is approximately

9 $$\ddot x\left( t \right) - \Delta gH\left( t \right) \approx - \Delta gH\left( t \right)\left[ {1 - \cos \left( {\frac{{2\pi t}}{{{T_R}}}} \right)} \right]$$

Where ${T_R} \approx 4R{\rm{/}}{c_s}$, $R{\rm{ }}$ is the hypocentral distance and ${c_S}$ is the shear wave velocity. If the gravity signal is changing with time as $\Delta g\left( t \right)$, then the expected gravimeter signal is

10 $$\ddot x\left( t \right) - \Delta g\left( t \right) \approx - \Delta \dot g\left( t \right) \ast H\left( t \right)\left[ {1 - \cos \left( {\frac{{\pi {c_S}t}}{{2R}}} \right)} \right]$$

Where $*$ is the convolution operator. For short times after the earthquake origin, a Taylor expansion of the cosine can be used to approximate (10) as

11 $$\begin{array}{l} 	 \ddot x\left( t \right) - \Delta g\left( t \right) \approx - \Delta \dot g\left( t \right) \ast H\left( t \right)2\pi {\left( {\frac{t}{{{T_0}}}} \right)^2} \\ 	 = \frac{{ - 2\pi }}{{T_R^2}}{{\int\!\!\!\!\!\int} {\Delta g\left( t \right)dt} ^2}\\ \end{array}$$

Therefore, the expected gravimeter signal for the Tohoku earthquake that is shown in Fig. 4a of Montagner et al.^1^, should be modified as

12 $${\rm{gravimeter}} \, {\rm{output}} \approx {10^{ - 3}}{{\int\!\!\!\!\!\int} {\Delta g\left( t \right)dt} ^2}$$

This means that the gravimeter response is very sluggish with time and it is poorly suited to rapidly detect earthquakes as is required for seismic early warning. Examination of Fig. 4b from Montagner et al.^1^ shows that their predicted gravitational signal significantly overpredicts the accelerations recorded by the gravimeter. Perhaps modification of their predicted acceleration with the convolution of (11) will produce better agreement with the observation.

Data sharing is not applicable to this article as no data sets were generated or analyzed during the current study.
